# Simultaneous analysis of multiple oligonucleotides by temperature-responsive chromatography using a poly(*N*-isopropylacrylamide)-based stationary phase

**DOI:** 10.1007/s00216-020-02749-8

**Published:** 2020-06-11

**Authors:** Yutaro Maekawa, Kaichi Yamazaki, Miwa Ihara, Kenichi Nagase, Hideko Kanazawa

**Affiliations:** Faculty of Pharmacy, Keio University, 1-5-30, Shibakoen, Minato-ku, Tokyo, 105-8512 Japan

**Keywords:** Green chromatographic system, Oligonucleotide, Phosphorothioated oligonucleotide, Simultaneous analysis, Temperature-responsive chromatography

## Abstract

Oligonucleotide therapeutics have contributed remarkably to healthcare, being well suited for the treatment of intractable diseases that are difficult to approach using conventional drug modalities. However, as common techniques of oligonucleotide analysis rely on reversed-phase or ion-exchange liquid chromatography and thus employ toxic organic solvents and/or ion-pairing reagents, better alternatives are highly sought after. Poly(*N*-isopropylacrylamide) (PNIPAAm) is widely used in temperature-responsive chromatography (TRC), which relies on column temperature variation to control the physical properties of the stationary phase and, unlike conventional reversed-phase liquid chromatography, avoids the use of toxic organic solvents and complicated gradient methods. Herein, PNIPAAm copolymer hydrogel-modified silica beads were used for the simultaneous analysis of multiple synthetic oligonucleotides by TRC to recognize differences in the length of single nucleotides, single bases, and the number of phosphorothioated sites. Temperature-responsive elution was observed in all cases. Each separation of all combinations of multiple oligonucleotides was better at higher temperatures above the lower critical solution temperature and was performed without the use of organic solvents and gradient methods. In the case of multiply phosphorothioated oligonucleotides, good separation was achieved using an aqueous solvent and isocratic elution in the absence of ion-pairing reagents. Thus, the developed procedure was concluded to be well suited for oligonucleotide analysis.

**Electronic supplementary material:**

The online version of this article (10.1007/s00216-020-02749-8) contains supplementary material, which is available to authorized users.

**Figure:**
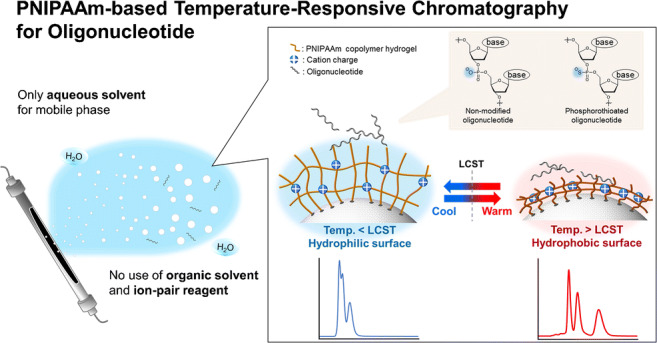
Graphical abstract

## Introduction

Oligonucleotide therapeutics have great potential for the treatment of diseases that are not easily approached using conventional drug modalities. However, only few of the numerous clinical trials of oligonucleotides satisfied their primary endpoint, which has, until recently, hindered further development in this field [1]. Today, oligonucleotide therapeutics have experienced a revival [1, 2] that is partly attributable to the better understanding of oligonucleotides and the recent progress in drug delivery systems [1]. The replacement of a non-bridging oxygen atom with sulfur in the oligonucleotide phosphate group affords a phosphorothioated oligonucleotide (S-oligo) with increased resistance to nuclease-catalyzed degradation and improved in vivo stability [3]. Synthetic oligonucleotides intended for use as medicinal products should be highly pure and free from length or sequence defects [4, 5]. In addition, the development of oligonucleotide therapeutics requires an evaluation of the structure and toxicity of impurities (oligonucleotide analogues) generated during oligonucleotide synthesis. Such impurities often have sequences similar to that of the target oligonucleotide, differing only in single nucleotide length, terminal single base, or the number of phosphorothioated sites [6]. Thus, the separation of the target therapeutic oligonucleotide from its analogues is essential from the viewpoints of quality control and safety [7, 8]. Currently, oligonucleotide analysis is commonly performed by reversed-phase liquid chromatography (RPLC) and ion-exchange liquid chromatography (IELC), which employ organic solvent-containing mobile phases and/or gradient methods [5]. In particular, several S-oligo analysis methods use ion-pairing reagents such as protonated triethylamine [9–11]. Generally, S-oligo molecules are more hydrophobic than their non-modified versions [10]. Besides, the asymmetry of the phosphorothioate phosphorus atom results in the presence of multiple stereoisomers and, hence, in substantial peak broadening [12]. A recent report showed that the peak broadening of S-oligos could be suppressed by using RPLC employing a mobile phase gradient containing organic solvent and ion-pairing reagent [11]. In view of these difficulties, few studies have explored S-oligo analysis without ion-pairing reagents.

High-performance liquid chromatography (HPLC) is widely used to determine the identity of organic compounds. In particular, RPLC, as the most common type of HPLC, enjoys high popularity but is characterized by excessive consumption of organic solvents, commonly generating more than 1 L of liquid waste each day for a single analysis [13, 14]. Hence, to minimize waste generation, green chromatography techniques have been put forward and researched [13–15].

Poly(*N*-isopropylacrylamide) (PNIPAAm) is one of the best-known temperature-responsive polymers, exhibiting a drastic temperature-dependent phase transition in aqueous media at a lower critical solution temperature (LCST) of approximately 32 °C [16, 17]. At temperatures below the LCST, the PNIPAAm chain is hydrated/hydrophilic; at higher temperatures, it becomes dehydrated/hydrophobic, which is widely exploited in fluorescent polymer probes [18, 19], drug delivery systems [20–22], microfluidics [23, 24], and cell sheets [25–27]. The aforementioned phase transition can be easily and sharply controlled only by temperature variation [28, 29]; moreover, the properties of PNIPAAm can be altered by introducing various co-monomers into the polymer unit [30, 31]. For example, when *n*-butyl methacrylate (BMA) is used as a co-monomer, hydrophobicity increases, while the introduction of protonatable-group-containing *N*,*N*-dimethylaminopropylacrylamide (DMAPAAm) enhances electrostatic interactions [32]. The application of these properties, observed for both PNIPAAm copolymer hydrogels and polymer chains [33], to chromatography allows one to achieve dramatic and reversible changes of surface hydrophilicity by simple variation of column temperature while avoiding the use of organic solvents [33–35], unlike for RPLC and IELC. Hence, temperature-responsive chromatography (TRC) is easier to perform and does not require the use of mobile phase gradients, as gradient analysis can be realized by simply changing the column temperature. Additionally, as the stationary phase becomes hydrophilic below the LCST, such columns can be washed with cold water only, while conventional RPLC columns need to be flushed with organic solvents. Thus, PNIPAAm-based TRC is expected to become a less complicated and environmentally friendly alternative to RPLC. The good performance of TRC using temperature gradients can be achieved with ordinary column ovens or water jacket owing to their great temperature response [36, 37]; furthermore, the performance of TRC will be maximized with a column oven that can change temperature more sharply and instantly.

Herein, PNIPAAm copolymer hydrogel-modified silica beads were used to develop a TRC method allowing one to recognize differences in the length of single nucleotides, terminal single bases, and the number of phosphorothioated sites. The proposed technique avoided the use of ion-pairing reagents, organic solvents, and mobile phase gradients, and was therefore well suited for securing the quality and safety of therapeutic oligonucleotides.

## Materials and methods

### Chemicals

*N*-Isopropylacrylamide (NIPAAm) and DMAPAAm were kindly provided by KJ Chemicals (Tokyo, Japan). NIPAAm was purified by recrystallization from *n*-hexane, and DMAPAAm was distilled before use. BMA, *N*,*N*′-methylenebisacrylamide (MBAAm), 4,4′-azobis(4-cyanovaleric acid) (V-501), and phosphate buffer (66.7 mM pH 7.0, 66.7 mM pH 6.6, and 66.7 mM pH 7.6) were obtained from Fujifilm Wako Pure Chemicals (Osaka, Japan). 1-Ethoxycarbonyl-2-ethoxy-1,2-dihydroquinoline (EEDQ) was purchased from Sigma-Aldrich (St. Louis, MO, USA). Aminopropyl-functionalized silica beads (pore size = 12 nm, average diameter = 5 μm) were procured from YMC (Kyoto, Japan). Deionized water purified by a Purelite PRB system (Organo Tokyo, Japan) was used to prepare the eluent and samples. All other chemicals were purchased from Fujifilm Wako Pure Chemicals. The synthetic oligonucleotides (Table 1), purchased from Tsukuba Oligo Service Corp. (Tsukuba, Japan), were deoxyribonucleosides.

**Table 1:** Sequences of oligonucleotides analyzed in this study

Name	Sequence	Number of bases	Concentration (μM)
dpT5	5′-d(TTTTT)-3′	5	54
dpT6	5′-d(TTTTTT)-3′	6	45
dpT10	5′-d(TTTTTTTTTT)-3′	10	54
dpT11	5′-d(TTTTTTTTTTT)-3′	11	49
dpT15	5′-d(TTTTTTTTTTTTTTT)-3′	15	65
dpT20	5′-d(TTTTTTTTTTTTTTTTTTTT)-3′	20	48
dCend	5′-d(CTCATCACAC)-3′	10	88
dTend	5′-d(TTCATCACAC)-3′	10	87
dGend	5′-d(GTCATCACAC)-3′	10	85
dAend	5′-d(ATCATCACAC)-3′	10	82
dpT10-7S	5′-d(T^T^T^T^T^T^T^TTT)-3′	10	81
dpT10-8S	5′-d(T^T^T^T^T^T^T^T^TT)-3′	10	81
dpT10-9S	5′-d(T^T^T^T^T^T^T^T^T^T)-3′	10	81
dpT15-12S	5′-d(T^T^T^T^T^T^T^T^T^T^T^T^TTT)-3′	15	54
dpT15-13S	5′-d(T^T^T^T^T^T^T^T^T^T^T^T^T^TT)-3′	15	54
dpT15-14S	5′-d(T^T^T^T^T^T^T^T^T^T^T^T^T^T^T)-3′	15	54

*d* deoxyribose, ^ phosphorothioated

### Synthesis and LCST determination of P(NIPAAm-*co*-BMA-*co*-DMAPAAm) linear polymer

The P(NIPAAm-*co*-BMA-*co*-DMAPAAm) (IBD) linear polymer was synthesized via radical polymerization as described elsewhere [33]. Briefly, NIPAAm (8.72 g, 77.0 mmol), BMA (0.60 g, 4.20 mmol), and DMAPAAm (0.67 g, 4.31 mmol) were dissolved in *N*,*N*-dimethylformamide (DMF) (80 mL), and the solution was then supplemented with 2,2′-azobis(isobutyronitrile) (radical initiator, 0.13 g, 0.81 mmol) and 3-mercaptopropionic acid (chain transfer agent, 0.21 g, 2.00 mmol). After 5-h heating at 70 °C, the solution was repeatedly poured into diethyl ether (300 mL) to precipitate the polymer as a white solid. The NIPAAm/BMA/DMAPAAm molar ratio was 90:5:5 and is hereinafter denoted as IB5D5.

The LCST of the IB5D5 linear polymer was determined from its optical transmittance. Specifically, 5 mg mL^−1^ solutions of this polymer in each mobile phase employed for analysis were subjected to measurements of optical transmittance at 500 nm using a UV–Vis spectrophotometer (V-630, Jasco, Tokyo, Japan). Solution temperature was controlled by a temperature controller (ETC-717, Jasco) and a PT-31 Peltier system (Krüss, Hamburg, Germany) at a heating rate of 0.1 °C min^−1^. The LCST was determined as the polymer solution temperature at 50% optical transmittance.

### Preparation of IBD hydrogel-modified silica beads

**Fig. 1:**
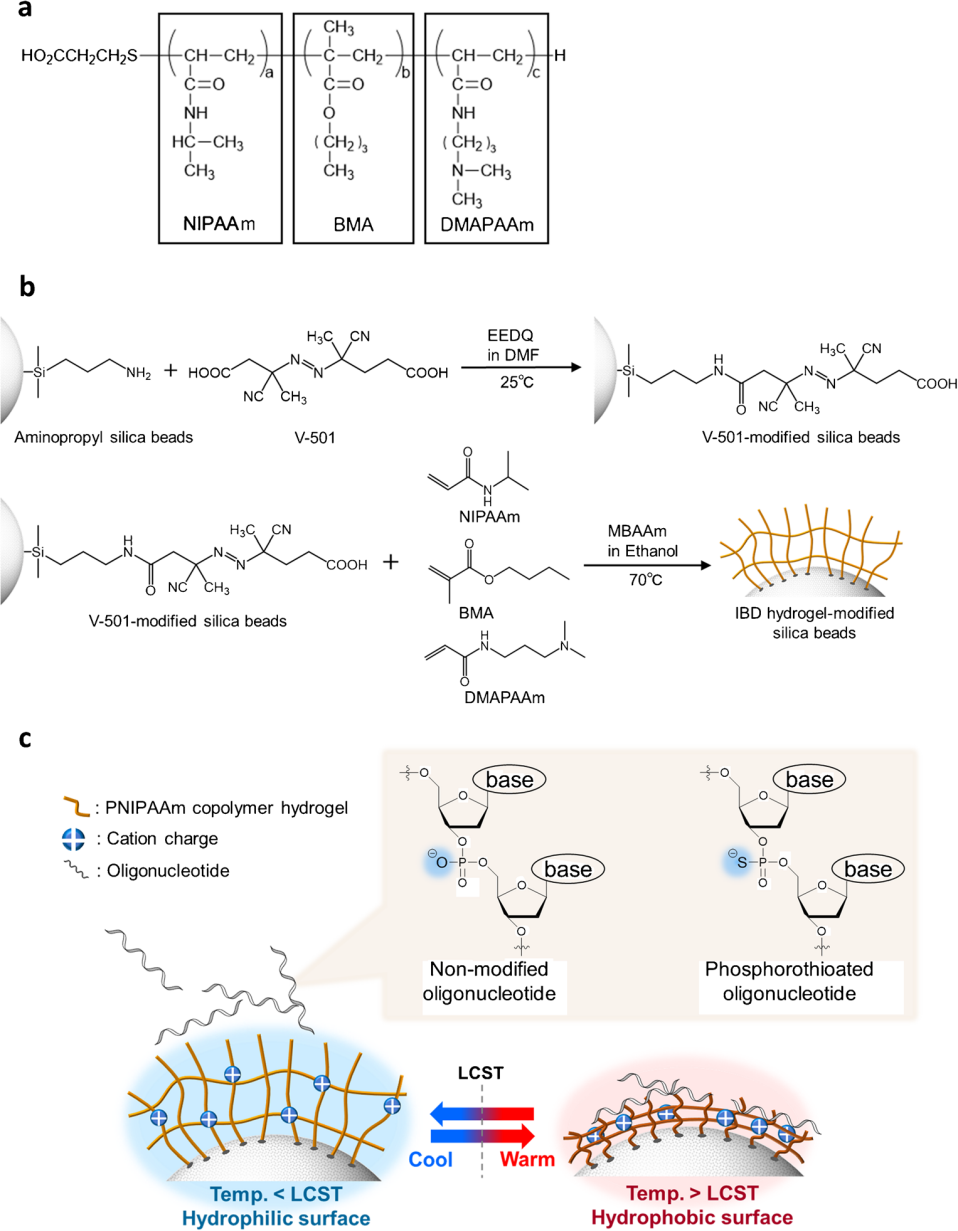
**a** Structure of IBD linear polymers, **b** synthesis of IBD hydrogels, and **c** concepts of oligonucleotide analysis using IBD hydrogel-modified silica beads

The ability of IBD hydrogels to separate multiple oligonucleotides was tested for four different compositions, namely for IB5D0 (NIPAAm/BMA/DMAPAAm = 95:5:0), IB5D1 (NIPAAm/BMA/DMAPAAm = 94:5:1), IB0D5 (NIPAAm/BMA/DMAPAAm = 95:0:5), and IB5D5 (NIPAAm/BMA/DMAPAAm = 90:5:5). IBD hydrogels were prepared and used to modify aminopropyl-functionalized silica beads as described elsewhere (Fig. 1) [34, 38]. Briefly, V-501 (initiator, 3.50 g, 12.5 mmol) and EEDQ (condensing agent, 6.18 g, 25.0 mmol) were dissolved in DMF (50 mL), and the solution was charged with aminopropyl-functionalized silica beads (5.00 g), and degassed by bubbling N_2_ gas for 30 min. The obtained mixture was shaken for 6 h at 25 °C, and the resulting V-501-modified silica beads were washed once with ethanol (500 mL) and dried in vacuo overnight. NIPAAm, BMA, DMAPAAm, and MBAAm were used as a temperature-responsive monomer, hydrophobic co-monomer, cationic co-monomer, and cross-linker, respectively. Typically, NIPAAm, BMA, DMAPAAm, and/or MBAAm were dissolved in ethanol, and the solution was charged with V-501-modified silica beads. The reaction mixture was degassed by bubbling with N_2_ gas for 30 min and heated at 70 °C for 5 h. The IBD hydrogel-modified silica beads were filtered, washed with methanol to remove non-immobilized hydrogel, dried in vacuo overnight, and packed into a stainless-steel column (50 mm length × 4.6 mm i.d. or 100 mm length × 4.6 mm i.d.). The following reagent quantities were used:

- IB5D0 hydrogel: NIPAAm (2.34 g, 20.7 mmol), BMA (0.16 g, 1.12 mmol), MBAAm (0.067 g, 0.44 mmol), ethanol (50 mL), beads (1.00 g).
- IB5D1 hydrogel: NIPAAm (4.16 g, 36.7 mmol), BMA (0.28 g, 1.97 mmol), DMAPAAm (0.061 g, 0.39 mmol), MBAAm (0.12 g, 0.78 mmol), ethanol (90 mL), beads (1.80 g).
- IB0D5 hydrogel: NIPAAm (4.66 g, 41.1 mmol), DMAPAAm (0.34 g, 2.18 mmol), MBAAm (0.14 g, 0.91 mmol), ethanol (100 mL), beads (2.00 g).
- IB5D5 hydrogel: NIPAAm (4.36 g, 38.5 mmol), BMA (0.30 g, 2.11 mmol), DMAPAAm (0.34 g, 2.18 mmol), MBAAm (0.14 g, 0.91 mmol), ethanol (100 mL), beads (2.00 g).

### Characterization of IBD hydrogel-modified silica beads

IBD hydrogel-modified silica beads were probed by elemental analysis, Fourier transform infrared (FTIR) spectroscopy, scanning electron microscopy (SEM), and zeta potential. The compositions of modified silica beads were determined by elemental analysis (PE2400 CHN elemental analyzer, PerkinElmer, Waltham, MA, USA), and attenuated total reflection FTIR spectra (FTIR-4700; Jasco, Tokyo, Japan) were recorded to confirm bead modification with each IBD hydrogel. Field-emission scanning electron microscopy (S4700, Hitachi High Technologies, Tokyo, Japan) was used to observe the surface morphology of IB5D5 hydrogel-modified silica beads. The zeta potentials of IB5D0, IB5D1, and IB5D5 hydrogel-modified silica beads were measured by laser Doppler velocimetry using a capillary cell (Zetasizer nanoZS, Malvern Instruments, Malvern, UK) to probe the electrostatic properties due to the presence of DMAPAAm. Detailed procedures for elemental analysis and zeta potential were described in Electronic Supplementary Material (ESM).

#### Performance evaluation of IBD hydrogel columns for the separation of multiple oligonucleotides

Each IBD hydrogel column (50 mm length × 4.6 mm i.d.) was connected to an HPLC system (Prominence-i LC-2030C 3D, Shimadzu, Kyoto, Japan). Column temperature was controlled by an SSC-2320 column oven (Senshu Scientific, Tokyo, Japan), and the detection wavelength was 260 nm. A 66.7 mM pH 7.0 phosphate buffer was used as the mobile phase at a flow rate of 1.0 mL min^−1^. Separation performance was tested using a solution of dpT10 and dpT11 in the aforementioned phosphate buffer.

#### Separation of oligonucleotides on IBD hydrogel columns by TRC

The prepared column (50 mm length × 4.6 mm i.d. or 100 mm length × 4.6 mm i.d.) was connected to the HPLC system. The 100-mm-long column was only used to separate oligonucleotides with different terminal single bases, while the shorter (50-mm-long) column was used for other separations. Column temperature and detection wavelength were as mentioned above. The separation of oligonucleotides with different length, terminal single base, and number of phosphorothioated sites was performed using 66.7 mM pH 7.0, 66.7 mM pH 6.6, and 250 mM pH 7.6 (containing phosphate [66.7 mM] and NaCl [183.3 mM]) phosphate buffers, respectively, at a flow rate of 1.0 mL min^−1^. All analytes were dissolved in 66.7 mM pH 7.0 phosphate buffer to the concentrations listed in Table 1.

## Results and discussion

### Characterization of IBD hydrogel-modified silica beads

The loading of each IBD hydrogel on silica beads was determined by elemental analysis (Table 2). The carbon content of V-501-modified silica beads was larger than that of non-modified silica beads, which was indicative of successful initiator grafting. The carbon contents of all IBD hydrogel-modified silica beads exceeded that of V-501-modified silica beads. The same trend was observed for nitrogen content except for IB5D0 hydrogel-modified silica beads, which was ascribed to the absence of DMAPAAm, unlike in the case of other hydrogels. These observations indicated that radical polymerization resulted in the successful grafting of IBD hydrogels onto silica beads.

**Table 2:** Elemental analysis of IBD hydrogel-modified silica beads

Modification	NIPAAm/BMA/DMAPAAm, mol/mol/mol	Elemental composition (%)		%C(calcd)^b^	Modified initiator (mmol m^−2^)^c^	Grafted hydrogel (mg m^−2^)^c^
		C^a^	N^a^			
None		3.23 ± 0.05	1.02 ± 0.02			
V-501		8.97 ± 0.03	3.19 ± 0.02	51.4	0.406	
IB5D0 hydrogel	95:5:0	14.3 ± 0.02	3.18 ± 0.02	63.9		0.333
IB5D1 hydrogel	94:5:1	15.7 ± 1.19	3.66 ± 0.23	63.8		0.435
IB0D5 hydrogel	95:0:5	15.4 ± 1.59	3.88 ± 0.38	63.5		0.418
IB5D5 hydrogel	90:5:5	14.7 ± 0.09	3.32 ± 0.03	63.7		0.366

^a^Determined by elemental analysis

^b^Calculated using the ratio of the molecular weight of carbon in each monomer to the total molecular weight of each monomer

^c^Estimated from carbon content

Figure S1 in ESM presents the FTIR spectra of hydrogel-modified silica beads, showing that peaks at approximately 1550 cm^−1^ (N–H bending vibration) and 1650 cm^−1^ (C=O stretching vibration) were observed for IB5D0, IB5D1, IB0D5, and IB5D5 hydrogel-modified beads. Conversely, weak absorptions were observed for V-501-modified silica beads, and no absorptions were observed for non-modified silica beads. Figure S2 in ESM presents SEM images of IB5D5 hydrogel-modified and non-modified silica beads, revealing that hydrogel grafting did not noticeably damage silica or affect the size of silica beads, i.e., controlled polymerization and modification were achieved.

**Fig. 2:**
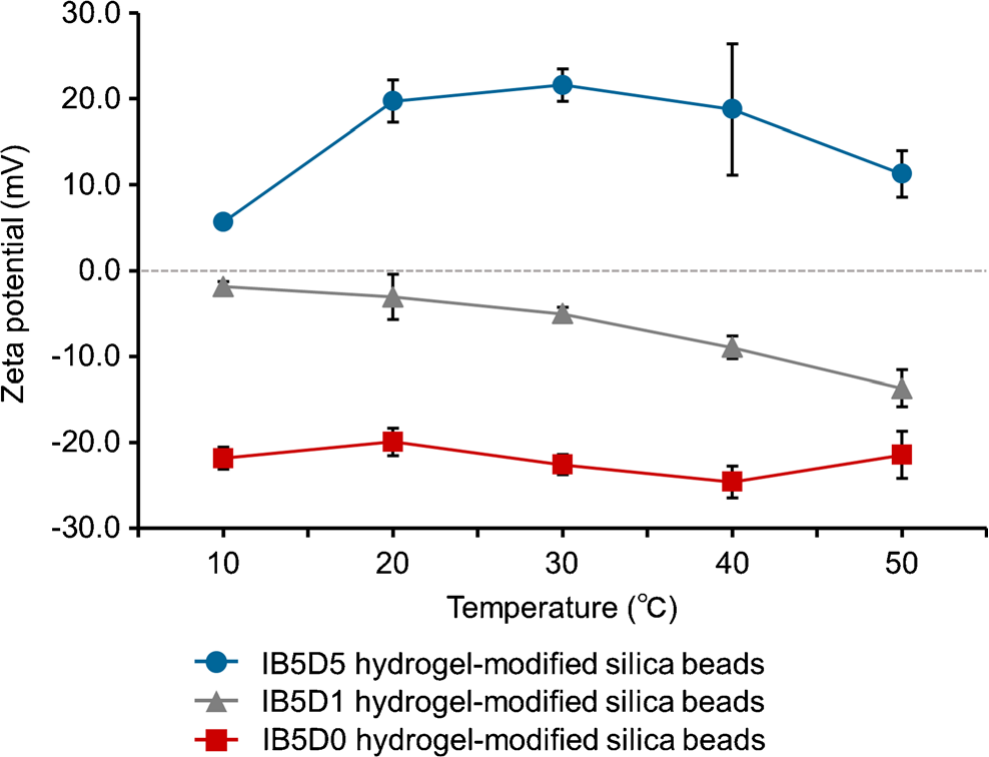
Temperature-dependent zeta potentials of IB5D0, IB5D1, and IB5D5 hydrogel-modified silica beads

The zeta potentials of IB5D0, IB5D1, and IB5D5 hydrogel-modified silica beads were indicative of a positively charged surface (Fig. 2), significantly increasing with increasing DMAPAAm content and slightly decreasing with increasing temperature. Thus, the PNIPAAm copolymer hydrogel-modified silica beads containing BMA and DMAPAAm as co-monomers exhibited temperature responsiveness and cationic nature (Fig. 1c).

### Evaluation of IBD hydrogel columns

**Fig. 3:**
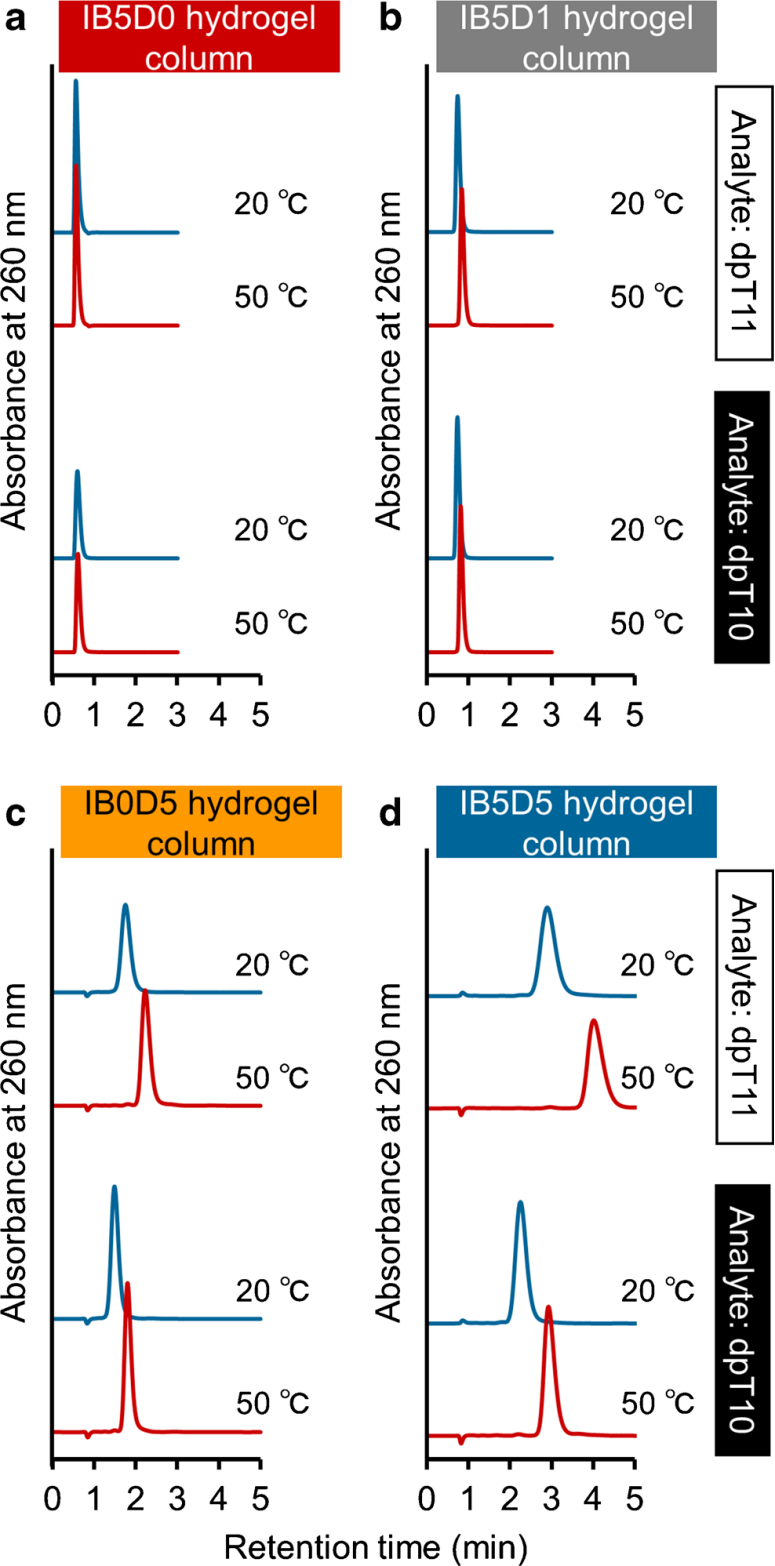
Chromatograms of dpT10 and dpT11 obtained for each column at temperatures of 20 and 50 °C

**Fig. 4:**
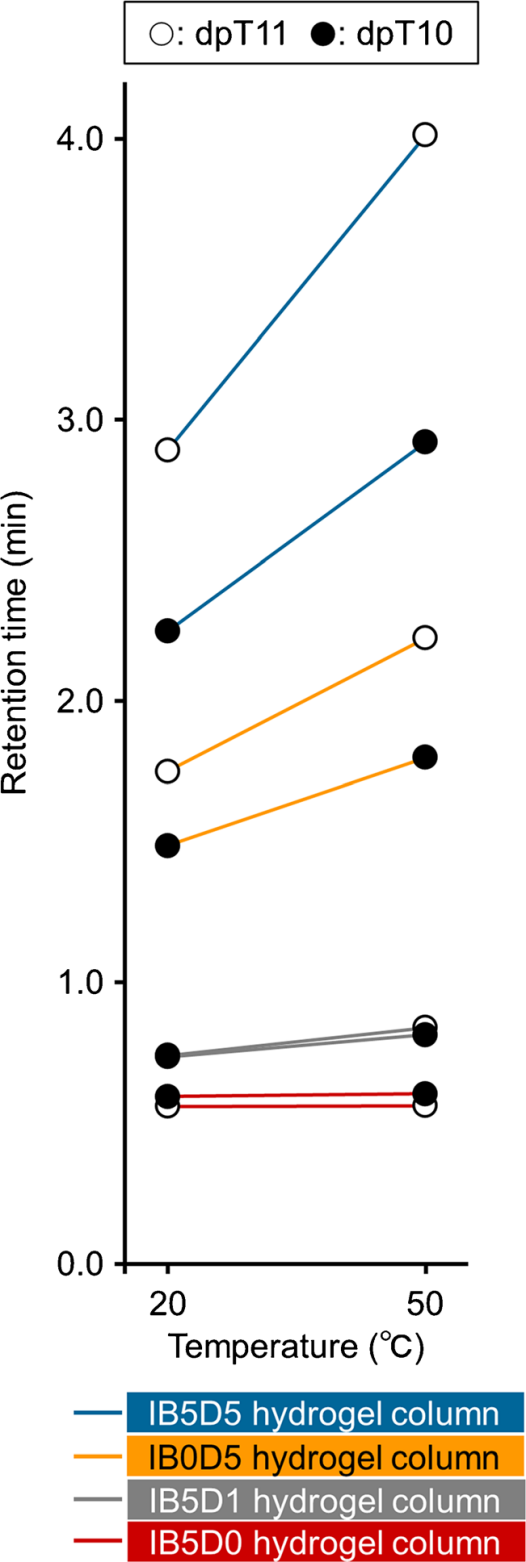
Comparison of dpT10 and dpT11 retention times for each IBD hydrogel column

The separation abilities of IB5D0, IB5D1, IB0D5, and IB5D5 hydrogel columns for multiple oligonucleotide analysis were evaluated using dpT10 and dpT11. Figure 3 shows the chromatograms of each analyte obtained at column temperatures of 20 and 50 °C, revealing that no or little retention was observed for IB5D0 and IB5D1 hydrogel columns. Conversely, IB0D5 and IB5D5 hydrogel columns engaged in stronger interactions with the aforementioned analytes and exhibited a temperature response, with the latter column featuring better separation at each temperature than the former (Fig. 4). Thus, for oligonucleotide separation, electrostatic interactions due to DMAPAAm were more essential than hydrophobic interactions due to BMA. This is consistent with previous reports concluding that an electrostatic interaction dominated the retention [39, 40]. Moreover, the observations suggested that (i) DMAPAAm acted as an ion-pairing reagent in the stationary phase and (ii) hydrophobic interactions due to BMA and the phase transition due to NIPAAm affected analytes interacting with DMAPAAm in the stationary phase. Thus, the IB5D5 hydrogel column was used for further analyses.

### Temperature response of IB5D5 linear polymer

**Fig. 5:**
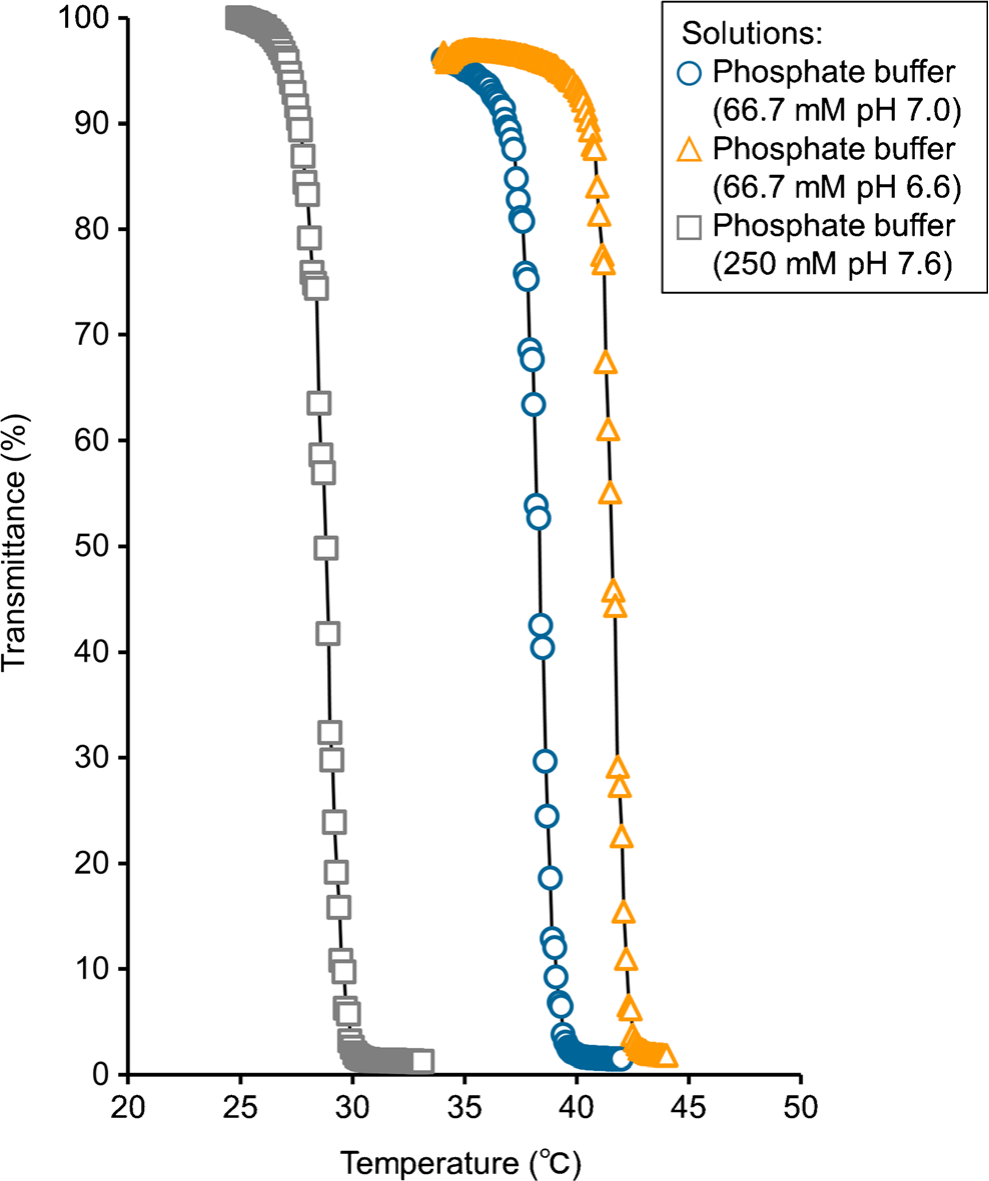
Optical transmittance–temperature plots used to determine the LCSTs of IB5D5 linear polymer in several media

Figures 1a and 5 show the structure of the IB5D5 linear polymer and transmittance–temperature plots used to determine its LCST in different solutions, respectively. Notably, LCST varied with pH and salt concentration, decreasing in the order of 66.7 mM pH 6.6 > 66.7 mM pH 7.0 > 250 mM pH 7.6, which was ascribed to the effects of these parameters on the protonation of DMAPAAm moieties [32]. That is, with increasing pH or salt concentration of the mobile phase, the dimethylamino moieties of DMAPAAm were increasingly deprotonated or screened with anions, which, in turn, increased hydrophobicity and decreased LCST. The IB5D5 hydrogel grafted onto silica beads was expected to have an LCST similar to or lower than that of the IB5D5 linear polymer, as this hydrogel was less flexible than the linear polymer and, hence, less prone to the NIPAAm phase transition.

### Separation of oligonucleotides with different lengths and terminal single bases

**Fig. 6:**
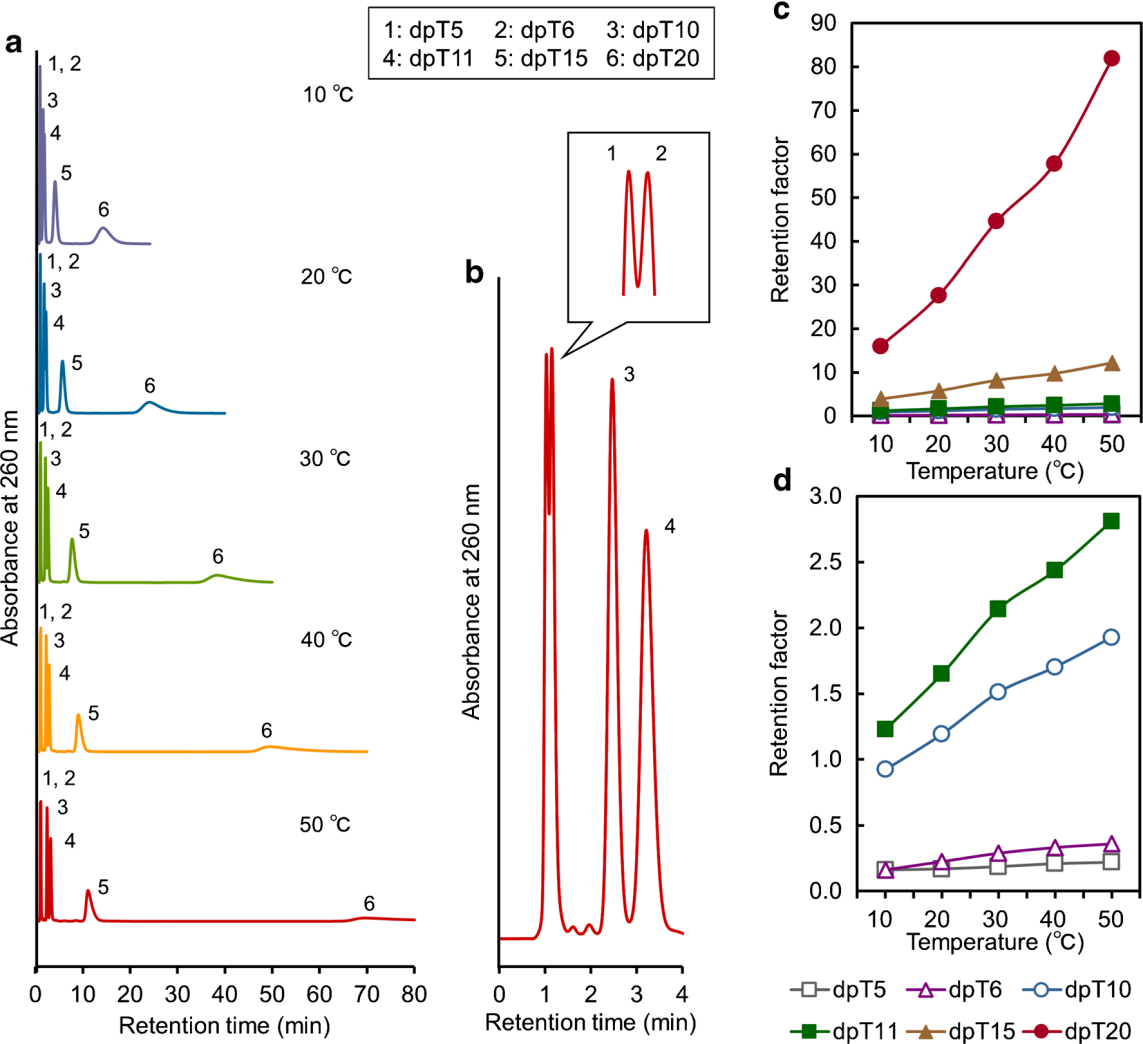
Chromatograms and retention factors of a mixture of multiple oligonucleotides with different lengths at various temperatures (**a** chromatograms at 10–50 °C, **b** chromatogram on enlarged scale at 50 °C, **c** retention factors, and **d** retention factors on enlarged scale)

**Fig. 7:**
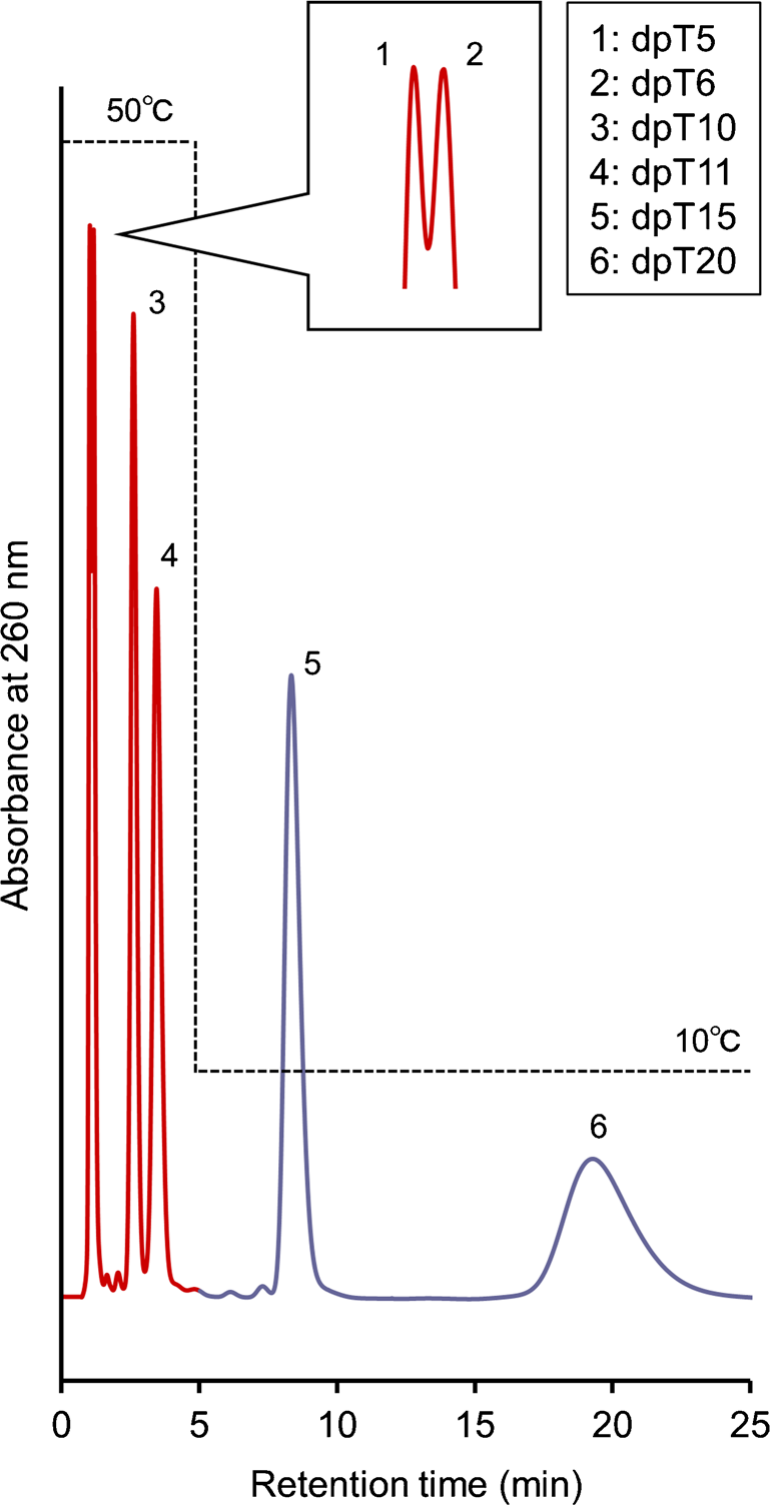
Chromatogram of a mixture of multiple oligonucleotides with different lengths obtained using a temperature step gradient

The elution behavior of six oligonucleotides with different lengths (dpT5, dpT6, dpT10, dpT11, dpT15, and dpT20) was observed for the IB5D5 hydrogel column at various column temperatures using 66.7 mM pH 7.0 phosphate buffer as the mobile phase (Fig. 6a). At 50 °C, analytes other than dpT5 and dpT6 were well separated, while the separation of dpT5 and dpT6 was small (Fig. 6c and d) and only observed at the peak tips (Fig. 6b). However, these two species could be better separated by a longer column. The temperature response was more noticeable for longer-chain analytes, as longer oligonucleotides feature stronger hydrophobicity and better ionizable phosphate groups than shorter ones and more easily interact with BMA (hydrophobically) and DMAPAAm (electrostatically). Previous study using RPLC and ion-pairing reagent showed that stationary phase was less selective for the longer oligonucleotides than for the shorter ones [11]. This difference would be derived from the two characteristics (hydrophobic and electrostatic properties) of IB5D5 hydrogel column. The longer oligonucleotides interacted for longer and more strongly with the stationary phase. Interestingly, all analytes were separated and eluted within 25 min when a temperature step gradient from 50 to 10 °C was used (Fig. 7), whereas at a constant temperature of 50 °C, separation took more than 80 min. The retention of dpT15 and dpT20, which were more strongly held at 50 °C than at 10 °C, was drastically weakened by the rapid phase transition of the stationary phase across the LCST.

**Fig. 8:**
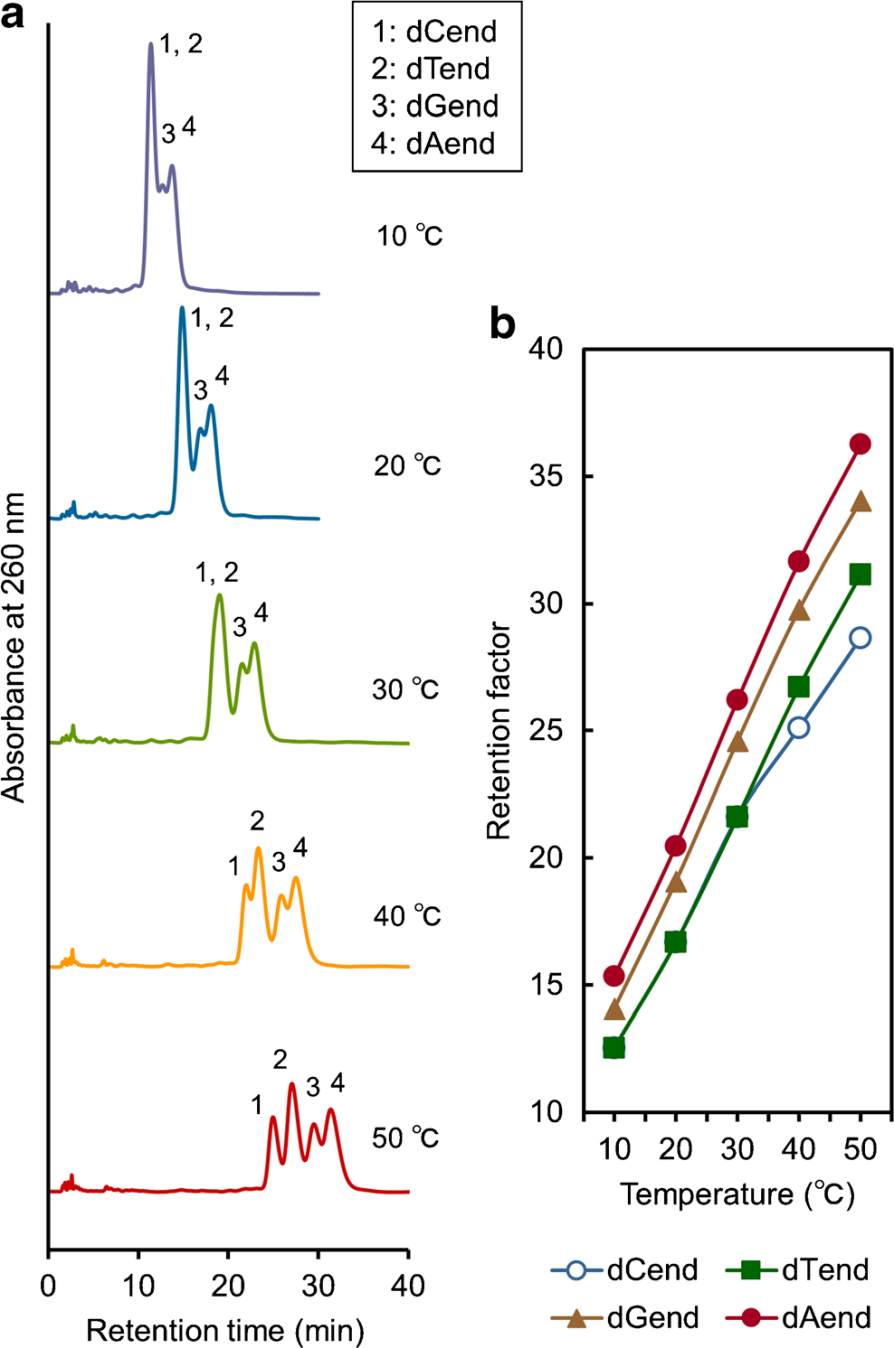
Chromatograms and retention factors of a mixture of multiple oligonucleotides with different terminal single bases obtained at various temperatures (**a** chromatograms at 10–50 °C and **b** retention factors)

Four oligonucleotides with different terminal single bases (dCend, dTend, dGend, and dAend) were analyzed by TRC using the IB5D5 hydrogel column and 66.7 mM pH 6.6 phosphate buffer as the mobile phase (Fig. 8). All analytes were affected by alternation of column temperature and were best separated at a column temperature of 50 °C among the investigated temperatures as shown in Fig. 8b; generally, better separation could be observed with a longer column. The difference of terminal single base can affect retention by the stationary phase. Although nucleobase hydrophobicity increases in the order of C < G < A < T [41], the elution order was dCend < dTend < dGend < dAend, i.e., dTend was less strongly retained than dGend and dAend, in disagreement with the hydrophobicity order. As the employed IBD hydrogel column exhibits temperature responsiveness (due to the transition between hydrophilic and hydrophobic phases) and positive charge, the elution order was affected by both of these characteristics. The above findings also suggested that the terminal single base affects the electrostatic properties of the whole oligonucleotide.

The employed method allowed us to separate six oligonucleotides differing in length and terminal single base. Importantly, this separation was achieved using an aqueous solvent and isocratic elution, not requiring a complex mobile phase gradient, while the use of a simple temperature step gradient that changed only the column temperature significantly reduced analysis time. These features of TRC due to the presence of PNIPAAm copolymer hydrogel-modified silica beads as the stationary phase are not observed for conventional RPLC and IELC.

### Separation of S-oligos with different numbers of phosphorothioated sites

**Fig. 9:**
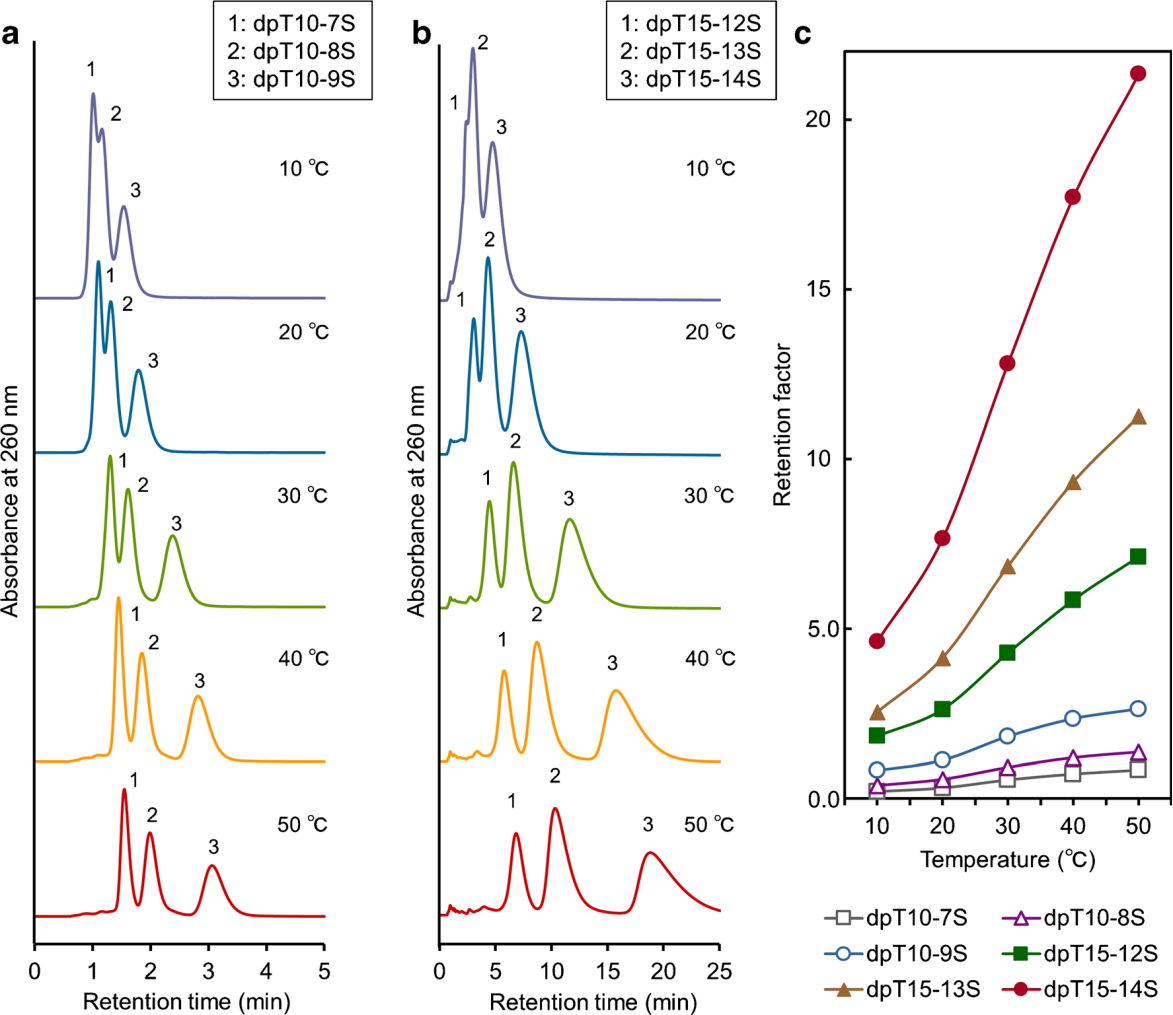
Chromatograms and retention factors of a mixture of multiple S-oligos with different numbers of phosphorothioated sites obtained at various temperatures (**a** chromatograms of 10-mer S-oligos, **b** chromatograms of 15-mer S-oligos, and **c** retention factors)

Three of the S-oligos with 10 and 15 residues and different numbers of phosphorothioated sites (dpT10-7S, dpT10-8S, and dpT10-9S for 10-mer S-oligos mixture, dpT15-12S, dpT15-13S, and dpT15-14S for 15-mer S-oligos mixture) were simultaneously analyzed using 250 mM phosphate buffer (pH 7.6) as the mobile phase and the IB5D5 hydrogel column. Good separation of both mixtures was achieved using only an aqueous solvent and isocratic elution (Fig. 9), i.e., in the absence of organic solvents and ion-pairing reagents. Oligomer hydrophobicity and number of stereoisomers increase with increasing number of phosphorothioated sites [10, 12], and so does the strength of the stationary phase–oligomer interaction. Hence, previous works employed ion-pairing reagents to separate S-oligos [9–11] and could not avoid the use of these reagents to discriminate between analytes differing by a single phosphorothioated site. In contrast, we could successfully analyze multiple S-oligos without using any ion-pairing reagents by TRC on an IB5D5 hydrogel column, as S-oligos moderately interacted with the stationary phase. The stationary phase of the IB5D5 hydrogel column was more hydrophilic than that of RPLC and featured a moderate positive charge due to the presence of DMAPAAm. Thus, the interaction between S-oligos and the stationary phase was not stronger than that in the case of RPLC, but also not overly weak to preclude retention.

Overall, the developed method allowed the separation of S-oligos with different numbers of phosphorothioated sites using only an aqueous solvent in the absence of ion-pairing reagents, organic solvents, and complicated mobile phase gradients, thus being sufficiently simple, unlike conventionally employed liquid chromatography techniques.

## Conclusion

A new method of multiple oligonucleotide analysis by temperature-responsive chromatography with PNIPAAm copolymer (IB5D5) hydrogel-modified silica beads as the stationary phase was developed, presenting a viable alternative to conventional techniques. All combinations of multiple oligonucleotides with differences in single nucleotide length, terminal single base, or the number of phosphorothioated sites were partially or well separated; importantly, good separation of multiple S-oligos was achieved using an aqueous mobile phase and isocratic elution. Additionally, the developed method avoided the use of ion-pairing reagents, organic solvents, and complicated mobile phase gradients, unlike RPLC or IELC techniques.

## Electronic supplementary material

ESM 1 (DOCX 1.96 mb)

## Data Availability

Data acquired in the current study are available from the corresponding author on reasonable request.
