# Supplementary material for: Simultaneous analysis of multiple oligonucleotides by temperature-responsive chromatography using a poly(N-isopropylacrylamide)-based stationary phase
Source: Anal Bioanal Chem. 2020 Jun 11;412(22):5341–51. doi: 10.1007/s00216-020-02749-8 (PMC7387324; doi:10.1007/s00216-020-02749-8)

Electronic Supplementary Material

Journal: *Analytical and Bioanalytical Chemistry*

**Simultaneous analysis of multiple oligonucleotides by temperature-responsive chromatography using a poly(*N*-isopropylacrylamide)-based stationary phase**

Yutaro Maekawa, Kaichi Yamazaki, Miwa Ihara, Kenichi Nagase, Hideko Kanazawa*

Faculty of Pharmacy, Keio University, 1-5-30, Shibakoen, Minato-ku, Tokyo 105-8512, Japan

*Corresponding author. Tel.: +81-3-5400-2657, fax: +81-3-5400-1378, e-mail: kanazawa-hd@pha.keio.ac.jp

**Supplementary Materials and methods**

*Characterization of IBD hydrogel–modified silica beads*

Elemental analysis was performed to determine the compositions of modified silica beads. The modified initiator (V-501) content of silica beads was assessed as

Modified initiator content = %C_i_ × [%C_i_(calcd) × (1 − %C_i_/%C_i_(calcd)) × *S*]^−1^,

where %C_i_ is the difference between the percentage of carbon in non-modified and modified silica beads; %C_i_(calcd) is the calculated percentage of carbon in modified silica beads, and *S* is the specific surface area of silica beads (310 m^2^ g^−1^).

The content of hydrogel grafted onto silica beads was calculated as

Grafted hydrogel content = %C_h_ × [%C_h_(calcd) × (1 − %C_h_/%C_h_(calcd) − %C_i_/%C_i_(calcd)) × *S*]^−1^,

where %C_h_ is the difference between the percentage of carbon in V-501-modified silica beads and IBD hydrogel–grafted V-501-modified silica beads, and %C_h_(calcd) is the calculated percentage of carbon in IBD hydrogel–modified silica beads.

The zeta potentials of hydrogel–modified silica beads were used to probe the electrostatic properties due to the presence of DMAPAAm. For measuring the zeta potential, the beads were suspended in 10 mM KCl solution at a concentration of 1.0 mg mL^−1^, and measurements were performed in triplicate for each temperature.

**Supplementary Figures**

**Fig. S1** FT-IR spectra of IBD hydrogel–modified and non-modified silica beads


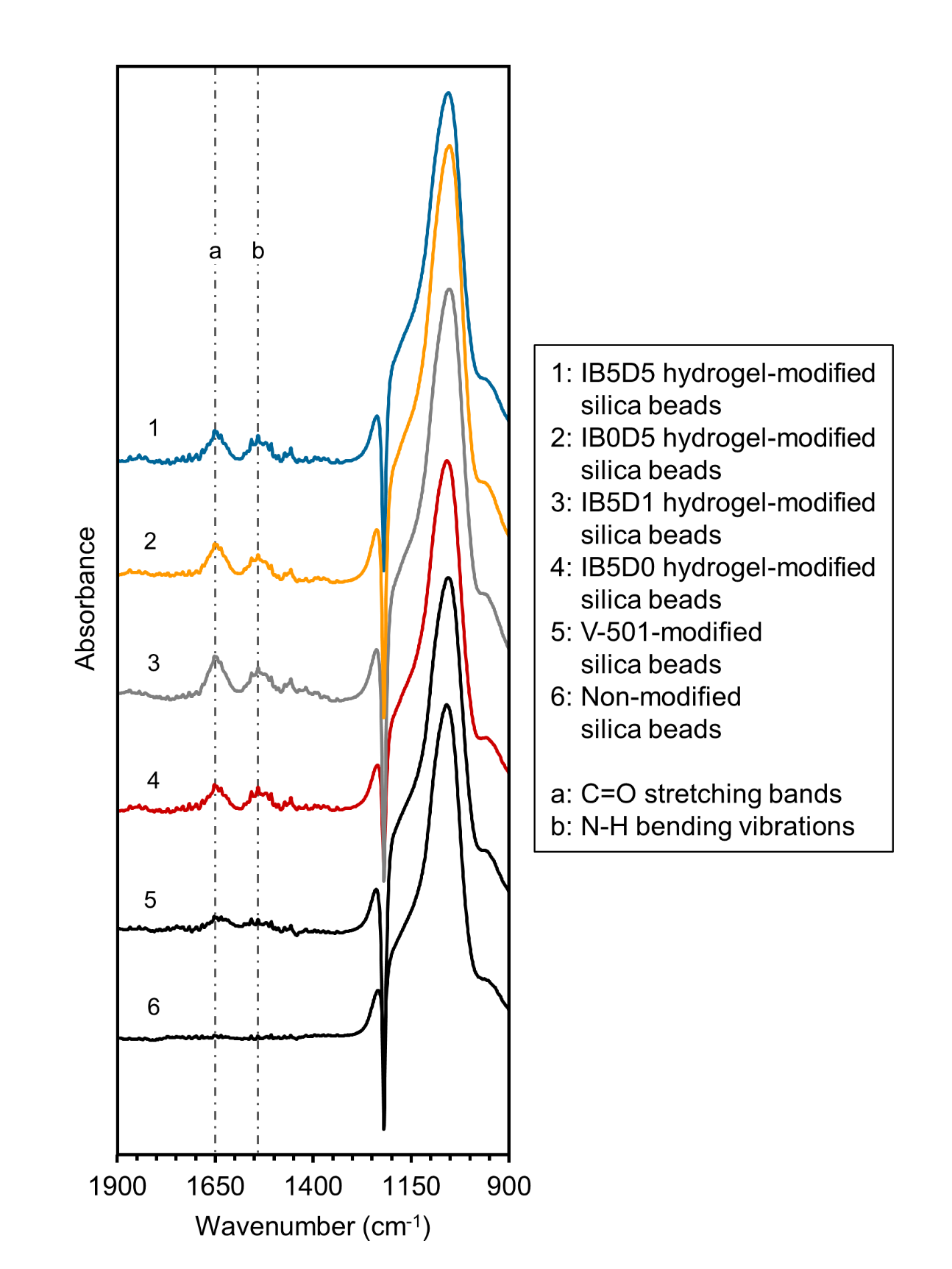


**Fig. S2** SEM images of (a) non-modified and (b) IB5D5 hydrogel–modified silica beads


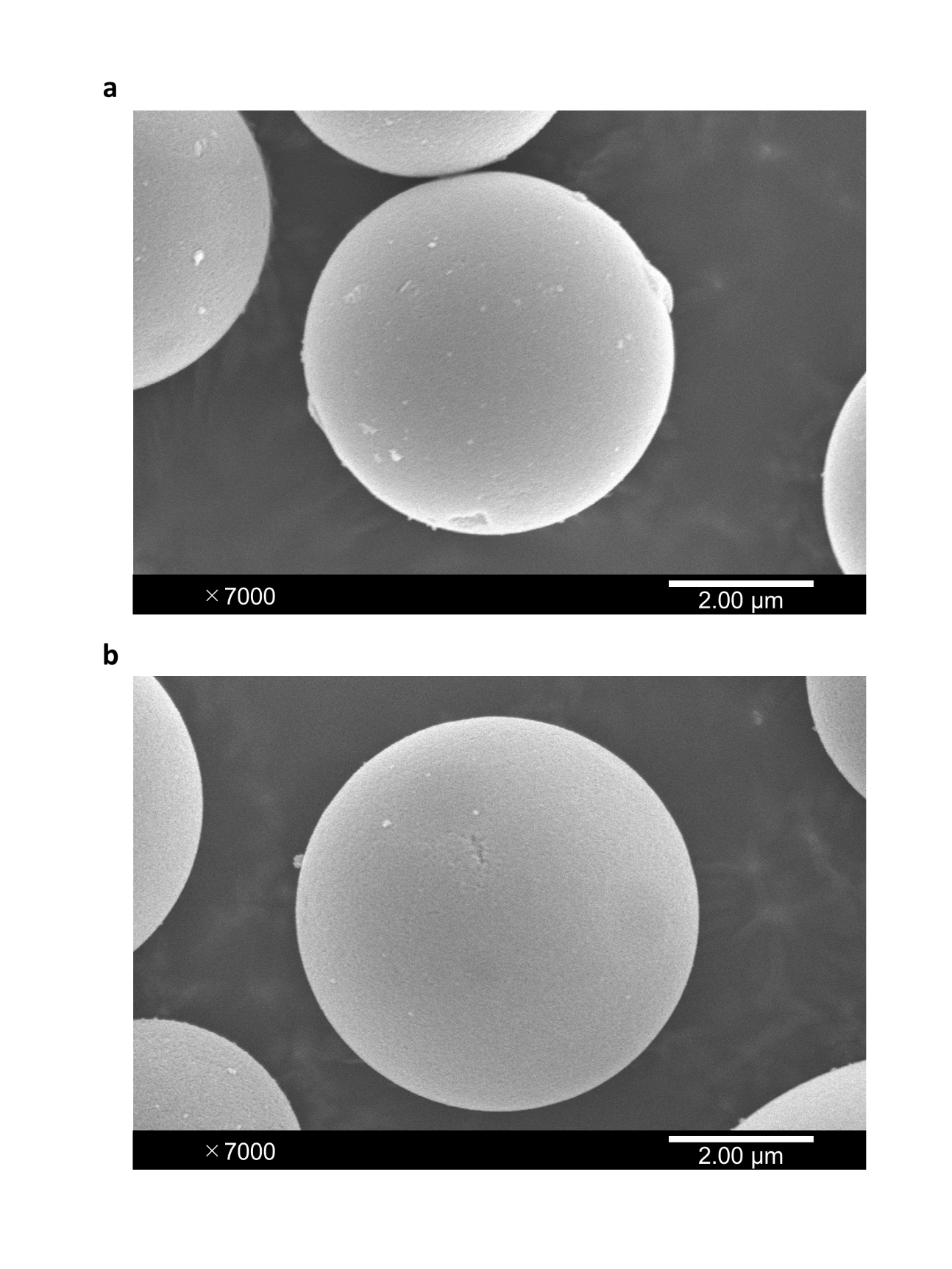

Supplement: Supplementary file 1 — (DOCX 1.96 mb) [file 216_2020_2749_MOESM1_ESM.docx]
